# How do mothers feel? Life with children with congenital Zika syndrome

**DOI:** 10.1002/ijgo.13044

**Published:** 2020-01-23

**Authors:** Paula S.S. Freitas, Gabriella B. Soares, Helaine J.S. Mocelin, Larissa C.X.L. Lamonato, Carolina M.M. Sales, Ana R. Linde‐Arias, Elda C.A. Bussinger, Ethel L.N. Maciel

**Affiliations:** ^1^ Laboratory of Epidemiology Federal University of Espírito Santo Vitória ES Brazil; ^2^ Graduate Program in Collective Health Federal University of Espírito Santo Vitória ES Brazil; ^3^ Department of Health Promotion Medical Sciences Center Federal University of Paraíba João Pessoa, Paraíba Brazil; ^4^ Health Sciences Department Federal University of Espírito Santo São Mateus ES Brazil; ^5^ Maurício Gastón Institute University of Massachusetts Boston MA USA; ^6^ Faculty of Law Graduate School of Law Vitória ES Brazil

**Keywords:** Brazil, Children with disabilities, Congenital Zika syndrome, Microcephaly, Mother–child relationships, Public Health, Zika virus

## Abstract

**Objective:**

To describe the repercussions, from the perspectives of caregiver mothers, of confirmed congenital Zika syndrome (CZS) in their offspring.

**Methods:**

A descriptive‐exploratory study with a qualitative approach was carried out in the state of Espírito Santo in southeastern Brazil, with 25 women who had a child diagnosed with CZS.

**Results:**

Emerging themes from the content analysis were grouped into two categories: (1) inequalities experienced by mothers, including social inequality, poverty, and gender inequality; (2) the impact of a child with CZS on mothering, including feelings at the time of diagnosis, maternal isolation and mental health, experiences of stigma and prejudice, and exhausting itineraries searching for therapeutic care.

**Conclusion:**

The repercussions of CZS were a huge burden on already vulnerable women, and social inequalities and poverty were important markers in the mothers’ reports. Many of the families affected by CZS already lived in precarious social conditions and these conditions were exacerbated further. Robust public and social policies to support these mothers need effective implementation given that babies born with CZS need long‐term care and support.

## Abstract 2

The repercussions of congenital Zika syndrome on mothers’ lives is a burden. Social inequality and poverty are important markers that exacerbate precarious social conditions.

## 1 INTRODUCTION

By the end of 2015, a large outbreak of Zika virus infection had been recognized in Brazil.^1^ At the beginning of the epidemic the infection was associated with numerous new cases of microcephaly. Subsequently, Zika virus infection was identified in the amniotic fluid of a pregnant Brazilian woman, and the fetus presented with microcephaly.^2^ In view of the association between Zika virus and fetal malformations, named congenital Zika syndrome (CZS), a public health emergency of international concern was declared.^3^

CZS is defined as the presence of microcephaly or other neurological, visual, motor, and hearing complications resulting from Zika virus infection.^4^ The syndrome strongly affected socioeconomically vulnerable communities, many of which are neglected by public authorities.^5^

In Brazil, 216 207 probable cases of Zika virus infection were reported in 2016; 17 594 in 2017; and 8024 in 2018. At the beginning of the outbreak, the reported suspected number of cases of altered child development related to Zika virus was 4121 in 2015; 8610 in 2016; 2653 in 2017; and 1657 in 2018, totaling 17 041 suspected cases. From week 45 of 2015 to week 52 of 2018, 2853 cases were confirmed, and those children were being supported by specialized childcare and early stimulation practices.^6^

The prevalence of live births with CZS was higher among mothers aged 24–40 years who were non‐white, had no higher education, were living in the northeastern region of the country, and reported to be single or living with a steady partner.^7^ Considering this background, these mothers and their children are exposed to a life of great difficulty. Special health policies are needed to address these matters, such as inclusive access to daycare centers and schools, multiprofessional monitoring, and special transportation for therapeutic care.^8^

Families of children living with CZS face demands for specialized care, as well as a lifelong responsibility for care.^9^ For mothers, an accumulated burden of factors influences daily care of children with significant disabilities. This burden must be discussed, including topics such as economic impact, lack of access to information and appropriate health care, prejudice, marital tension, limited family support, and uncertainty about the future for their children.

Few studies have discussed the issues surrounding the care of children with CZS or have given voice to women who deal daily with the epidemic. The aim of the present study was to describe, from the perspectives of caregiving mothers, the repercussions on their lives after confirmation of CZS in their child.

## 2 MATERIALS AND METHODS

We conducted a descriptive‐exploratory study with a qualitative approach in the state of Espírito Santo, southeastern Brazil, with 25 women who had a child with CZS. The state covers 46 095 km^2^ and is divided into 78 municipalities. It has a population of 4 016 356 inhabitants and is one of the few Brazilian states in which the state capital is not the most populous city.^10^

Researchers approached the Microcephaly Committee of the Health Department of the State of Espírito Santo to investigate cases that had occurred since the beginning of the outbreak (November 2015 until December 2016). During this period, there were 49 confirmed cases of newborns with CZS, including 10 spontaneous abortions or stillbirths.^11^ All 39 mothers of CZS newborns reported between 2015 and 2016 with confirmed diagnosis were invited to participate in the study and 25 agreed.

The reasons women (n=14) refused to participate or could not be included in the study were as follows: four women moved out of the state in search of better health care for their babies; two women were underage mothers who did not obtain parental consent to participate; one mother was living on the street and was not found; one mother gave up her child to a shelter and was deprived of parental power; the babies of two mothers died in 2017 due to complications of CZS; one mother's baby was hospitalized in the Pediatric Intensive Care Unit and she was unable to participate owing to emotional distress; and three mothers declined to give an interview.

For data collection, interviews were conducted using a semistructured script with the following guiding questions: “How was your pregnancy? How was your baby's birth? Tell me your experiences, perspectives, and feelings when caring for your child in your daily life? How has your life been after the birth of your child?” The interviews also collected sociodemographic, clinical, and obstetric history data from the participants, such as age, marital status, education, occupation, family income, social benefits, number of people in the family, and number of pregnancies. Data on details of arbovirus infection such as diagnosis of dengue, diagnosis of other cases of Zika in the family, use of repellent during pregnancy, presence of health insurance, and place and number of prenatal visits were also collected.

Interviews took place individually and were conducted between May 1 and September 29, 2017, audio‐recorded digitally, and authorized by the participants. All women chose their homes for the interviews; in addition to the digital recording device, a paper diary was also used for notes and observations captured during the encounters.

All of the interviews were transcribed and analyzed according to thematic content analysis. Thematic analysis follows a trajectory that consists of categorization, inference, description, and interpretation of data. Categorization requires great knowledge from the researcher to determine an adequate classification plan. Inference is made when something is logically deduced from the content being analyzed. Description is the listing of characteristics of the text, summarized after analytical treatment, and interpretation is the meaning given to these characteristics.^12^

The research project was approved by the Research Ethics Committee of the Health Sciences Center of the Federal University of Espírito Santo (CEP/CCS/UFES) under protocol number #1730.231/2016. It was also approved by the PAHO Ethics Review Committee (PAHOERC) under PAHO‐2017‐02‐0013. Interviews were conducted only after a signature confirming informed consent had been obtained from each participant. Participant anonymity was protected through identification using the letter “M” and respective numbers referring to the sequence of the transcription.

## 3 RESULTS

**Figure 1:**
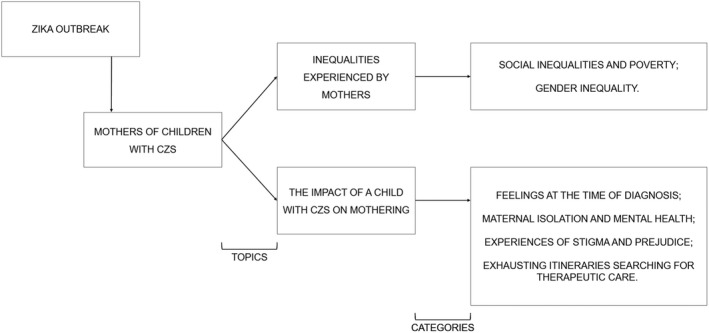
Matrix of the analysis.

After analyzing the data, two themes emerged to group the categories according to the analysis matrix (Fig. 1), namely: (1) inequalities experienced by mothers, including social inequality, poverty, and gender inequality; (2) the impact of a child with CZS on mothering, including feelings at the time of diagnosis, maternal isolation and mental health, experiences of stigma and prejudice, and exhausting itineraries searching for therapeutic care.

Although the main objective of the present article was not to outline the socioeconomic profile of the participants, this characterization allows an understanding of the backgrounds of these mothers. The socioeconomic profile of the participants was age between 18 and 39 years, most were involved in a steady relationship, were non‐white, with high school education, and were unemployed. They lived primarily in the metropolitan region of Espírito Santo in rented houses in the suburbs, under poor living conditions; they had more than one child, and income consisted only of social benefit, named continued benefit (BPC), granted by the Brazilian government of approximately one minimum wage.^13^, ^14^

### 3.1 Inequalities experienced by mothers

#### 3.1.1 Social inequalities and poverty

The profile of the interviewees showed that these women lived in poverty and faced socioeconomic difficulties and various vulnerabilities before the existence of CZS in their lives.

The struggle to survive, provide treatment, and meet the needs of the family—heightened by having a child with CZS—was clear in the narratives of many mothers, as observed in the statements below:

Early in my pregnancy I was going through a rather complicated phase because I only had the “bolsa familia” grant; my husband when he would find a sporadic job to do, he would do it, and that's what we were living with. Today my daughter still does not have the social benefit. We did not expect her to come into the financial situation we were in. For me, the fact that she has microcephaly is not what hits me the most […] The only thing that is sad for us here at home is to know that she was born and she lives in a very difficult financial situation. We do not have decent living conditions. M16

We spend a lot, as soon as I get the benefit, pay the rent, 550 reais, gas, water, food, transportation, special milk and treatments. I paid 2 cast changes, a total of 300 reais, the first doctor was 300 reais. People from the church were making food to sell, but even so I was not able to pay anymore. It is very difficult, it's a lot of money. I could not keep her treatment. We cannot work, it's hard, I starve with my daughter, I work, but I do not leave her with anyone, because nobody will take care of her as I do. M2

Precarious housing situations and unhealthy environmental conditions in which the mothers lived were identified, such as problems with garbage in inappropriate places, open sewage, poor basic sanitation, and consequently the presence of many mosquitoes. Some women emphasized that it was not easy to avoid mosquito bites.

Here, it was a focus, there was a lot of mosquitoes. Sometimes we were talking and saw the house full of mosquitoes. M6

There was always a lot of mosquitoes here because of the trash. M5

#### 3.1.2 Gender inequality

The interviews revealed that before the birth of the baby with CZS, several mothers used to work or study. These tasks were replaced by the care of the child.

I was a general service assistant, now a sales clerk. But if my son had given me the chance, I was going to work as a dental assistant. M6

I went up to seventh grade and I got pregnant too early, I had to quit when the baby was born. I want to go back to school, but now it's kind of difficult. M11

Coercion to quit a job or dismissal after the birth of the child with CZS was a constant in the narratives of the women. A painful and difficult choice between the routine of caring for their child and the responsibility to work marked their lives.

She said she was just firing me because the baby needed more care. I talked to her, “Why does my daughter have anything to do with it? She did not ask to be born like this!” I felt very embarrassed. So humiliated, offended, because of hearing this. M1

I was working in the call center. And I had to quit my job because of the baby's pneumonia because I was missing too much work. M13

I worked my entire pregnancy. Then I got the maternity leave, so I came back and they dismissed me. According to company rules, I could not take sick leaves, and my son was going to start doing physiotherapy. M17

I started to leave my son in the daycare, terrible daycare service. Wow, I would cry every time I left my son because they would not even shower him, they would not change diapers, and he got full of chafing. And I would arrive at the company and ask: “for God's sake, send me away.” M18

Unemployment and the unequal burden of caring for a child with CZS added to the narratives revealing marital infidelity, abandonment, and physical and verbal aggression by their partners.

I did not want to continue the relationship when I discovered that he had cheated on me, but I was pregnant. M13

He was assaulting me. I went to the police station and reported it. One day he came home and took a knife and said: “If I only dream that you're cheating on me […] I'll kill you and kill the baby too. And then I'll kill myself”. Out of fear, I left home wearing only my clothes. M17

Before we had the baby, everything was fine between us. But as the baby cried a lot, day and night because of microcephaly, he even had to move rooms so he would not hear him crying. He could not stand it and went away. M11

When she was born, we separated. During my pregnancy, we had a lot of fighting, he used a lot of drugs, he used to beat me, because he thought it was my fault the baby was born with microcephaly […] He would beat me a lot and I was hungry so that to survive I prostituted and had relationships with men. And he would stay at home, he did not want to work. I was not embarrassed; I was prostituting with older men to get the money to support my daughter. M8

Not only did partners blame the mothers for the child's illness, as described by M8, health professionals also judged them to be responsible for preventing Zika, as described:

I did not wear long sleeves and the repellent warmed my skin when it was time to go to work how was I going to use repellent all the time, how could I walk covered in the heat? I was supposed to use, but I did not use. M12

So, when I went to the clinic, the nurse turned to me there and said, “For God's sake, why did you get pregnant now?” I said: “Okay, but is it any problem for me to get pregnant now?” She said: “Because we have a mosquito problem there and we are in the focus area.” Then I said: “How so?” She said: “You're going to have to wear a lot of long sleeves, long pants and whatever, and repellent,” because Zika virus is this and that, it can happen this way, so with the baby, etc. I said, “My goodness!” M6

### 3.2 The impact of a child with CZS on mothering

#### 3.2.1 Feelings at the time of diagnosis

Mothers felt fear about fetal malformation and anxiety during pregnancy, which increased after diagnosis.

I was very scared! M25

My goodness, it felt like the world collapsed on my head. At first, I did not want to comment or talk about it, because it was very difficult for me. But then I started to accept. M22

At the time of the ultrasound, the doctor said to his secretary: “There is something strange here,” he said to her: “Call the other doctor there,” she called the other doctor, he came, then when I looked, three doctors inside the room. I said, “My goodness!” I started to get scared and cry. Then he said: “You don't need to be scared, calm down!” And the other went and said: “There is no way out! You can put it there at the conclusion of the exam, it is really microcephaly,” then kept perplexed… Then he said: “Good luck!” And I left crying. M23

Not all women received a diagnosis of CZS during pregnancy because it was an unknown disease and, as such, there were delays in prenatal diagnosis. Many received the traumatic news intrapartum or immediately postpartum, revealing the poor ability of health professionals to deliver difficult information in a sensitive way.

The doctor was the one who caused me a shock. The doctor said that she was not going to see, she was not going to walk, she was just going to be a vegetable child, that is, the rest of her life and that she would need me. So she gave me that shock, and I'll be honest, I wanted to “jump” over the doctor. M8

The doctor on my side saying, “You know your daughter has microcephaly.” I said: “No, I did not know.” Like that, I was still expecting hydrocephaly. Then she said: “Yes, she has it”. I said: “Wow, but it does not look like it.” “Oh yes she does, it looks like her head is small!” Then she turned her back and left. Like this. So, at the time I did not know if I should cry, because I was not expecting, or if I should fly on her neck. Come on, she could have been a bit more delicate, because it is a difficult time. M14

No one explained anything. And that's when the doctor came with a lot of academics there, the doctor there, right: “Look, this is a child, probably congenital Zika, she has microcephaly…” That's when I looked, like that, it was like that. Then I said: “Doctor, wait a minute, is it confirmed? Microcephaly?” She said, “It is.” But no one came and said: “Look, mother” […]. That's the way I heard it. They were there, talking to each other there, explaining each case, for them to learn. Then she turned and said it like that. “This is a child with microcephaly, malformation, calcification, congenital Zika,” such and such. So, I looked, there, kind of, oh my God. […]. There at that moment, I wanted to scream, I wanted to somehow pour this out, you know? M2

At the moment of childbirth, when he was already born, right, the doctor said: “Look, the news is sad. But I have to tell you; the baby was born with the little head. We are going to investigate for Zika” […] It's like, my first child. The situation he was born, like I said, I would look at him and faint, I could not stand to see him. And it was, like this, I don't know, very ugly thing for a mother to see, I did not expect that. And it was very sad for me. I did not want to see it. I screamed and cried. Even after the birth I cried a lot. M9

#### 3.2.2 Maternal isolation and mental health

After the shock of the diagnosis the mothers reported changes to their daily lives. The routine was solitary and tiring; feelings of invisibility and isolation were present in the reports.

I used to work, take care of myself, […] now I don't eat, I quit doing everything, I don't even go to the bathroom sometimes, for her not to cry. Because she has these nervous breakdowns and she cries a lot. I'm suffering a lot. I did not sleep this night. M2

My life has changed a lot with him. I used to go out a lot, I wouldn't stop at home. But now, I'm home alone. M9

I have no social life. I have a life with the baby. I don't go out, I'm very homely, I like to stay home with my family. If I go out, I go to my relatives’ and come back. M13

Reports of their changing life circumstances and the pressure on these women revealed important mental suffering and family disharmony.

After I had him, I already had to take controlled medication, the doctor said that it was to give me more courage because there are days when it seems that I cannot stand or get up, so tired! Even the psychologist has already said that I need to rest […] nobody knows how to take care of him only me. For me to leave is a disaster. Goodness, there's been two months that I go to the capital every week for his treatment, and my other son is revolted, he said that he did not want to have a brother anymore, because he says that after his brother was born “…this house has no peace anymore, there is no peace,” he says that no one cares for him anymore, because really the brother takes my whole time! It's night and day! M23

And so, tiredness strikes, that routine, I don't go out, I don't do anything, I live inside this house, at night awake or going too much to the hospital, doctor, we just need to talk sometimes, we just need to cry, be heard. I don't need anyone to tell me ‘cause it's not going to change anything. Because like I said, I suffer because my daughter will not be able to marry, will not be able to develop, to have a family, you know? M2

#### 3.2.3 Experiences of stigma and prejudice

Mothers reported being questioned about their child's physical appearance from people they encountered:

Inside the buses, people keep staring at her plastered, I'm afraid of being assaulted, because they might think I hurt her. It's horrible, people shout, “How did you break that girl's legs?” It's horrible. M2

Some people ask, “Wow, she's sick, right?” I said, “No, my daughter is not sick.” I have two sisters who used to come to my house all the time, now they don't come anymore, they think microcephaly is transmissible. M8

There are people who keep looking at the baby with curiosity and this bothers me. I'd rather they ask what's wrong with him, I'll answer them, but when they ask, “Wow, why does your son have a big head?” I don't accept that. And I don't accept when they ask if he's sick. M17

#### 3.2.4 Exhausting itineraries searching for therapeutic care

Care routines for their children were exhausting and the mothers’ time was exclusively dedicated to their children's needs, multiple medical consultations, and early stimulation activities. Visits to the Association of Parents and Friends of Exceptional People (APAE) and the Center for Physical Rehabilitation of the State of Espírito Santo (CREFES) were common.

Mothers living in municipalities far from the capital faced access problems as treatments were only offered in large urban centers. Transport from the municipalities took them to the city and returned them by the end of the day. The mothers reported extremely difficult therapeutic itineraries.

Our week is a rush. From Monday to Friday there are consultations. I think we stay more time on the bus than at home. M14

On Mondays and Wednesdays, I take him to physical therapy, we wake up at five o'clock in the morning to get the first bus, and around 22 o'clock I'm at home […] It's tiring, there's not a single day he does not have crisis of crying and irritability. M22

The transport of the municipality leaves from there two and a half in the morning and his consultation is at eight, and there are patients who have consultations four o'clock in the afternoon, and there's only one car for the whole municipality. M25

I was there in Vitória for the exams; the bus of the municipal health department took me, dropped me inside the hospital and did not come back for me. I did not have the money to go back to my city. M18

## 4 DISCUSSION

The results of this study reveal the challenges faced by women living in poverty‐stricken areas who have given up their lives to care for children directly affected by the Zika virus epidemic. Some of the mothers experienced domestic violence and were abandoned by their partner; they reported a lack of sensitivity and care skills by health professionals at the time of diagnosis; felt blamed for contracting Zika virus and responsible for family planning; and suffered from the stigma of having a child with CZS and exhausting therapeutic itineraries seeking care for their children.

The social inequality and poverty described by the women was exacerbated by having a child with CZS. Furthermore, a link between CZS and low socioeconomic status has been suggested. A recent study showed a lower prevalence rate of microcephaly among families from wealthier socioeconomic strata, showing a strong association between higher prevalence of microcephaly and low socioeconomic level.^15^ It is important to recognize that abortion is illegal in Brazil and families of low socioeconomic status face the greatest challenges in raising a disabled child. Furthermore, reproductive decisions are intimately related to personal convictions and cultural beliefs and are embedded in sociocultural norms.^16^

M16 reported feeling greater sadness caused by her family's miserable living conditions than her daughter's diagnosis of microcephaly associated with CZS. This reflects the results of a study reporting that primary caregivers of children with CZS present depressive symptoms when faced with economic challenges related to the child's delayed development.^16^ In other words, economic challenges have a greater influence on the mental health of primary caregivers than the daily care of the child. In the study by Santos and Farias,^17^ financial issues were revealed by mothers as a difficulty that generated anguish; moreover, the majority of families suffering from the consequences of Zika virus infection were economically vulnerable. These unfavorable socioeconomic conditions make it even more complicated for mothers to care for children affected by CZS, which may compromise the child's development.^7^

Vulnerable groups suffering from the burden of mosquito‐borne diseases, such as Zika, are excluded from formal jobs and fair wages, have poor quality of life, and their housing is characterized by unhealthy environmental conditions.^18^ In Pernambuco—the state greatest hit by the Zika epidemic—most affected families lived in extreme poverty and, for the most part, had no water supply or garbage collection and inadequate sewage and drainage.^19^ This type of environment promotes favorable conditions for the mosquito to reproduce. Therefore, taking care of the environment and investing in basic sanitation as a basic human right is the most effective way of eliminating mosquito breeding sites.^19^

Considering gender inequality, women in the present study experienced an unequal burden of childcare and job loss resulting from lack of a support network. Balancing a professional life and domestic responsibilities continues to be a problem experienced by the majority of women in Brazil. Several national surveys have shown that the number of hours that women spend doing domestic tasks, even while employed in paid work, is much higher than for men.^20^ In patriarchal cultures, such as Brazil, domestic work is considered the natural role for women.^20^ Caring for a child with CZS is an additional burden on women who are already overloaded. Faced with this situation, mothers tend to abandon their professional activities, which reduces the family income at a time when there are high costs associated with a child's treatment.^21^

In Brazil, those aged 25–29 years who are unemployed and not in education are primarily young mothers who have had to abandon work and study to take care of their children. Public policies that support women to reconcile work and mothering are lacking.^20^

In the present study, gender inequality also manifested as conjugal infidelity, abandonment, and physical and verbal aggression by partners. Similar data have been described where approximately half of the women affected by CZS in Brazil had no partner, were single, or separated.^22^ In an ethnographic study conducted in northeast Brazil, Diniz^23^ reported that abandonment of spouses and partners after learning that the baby had neurological problems was common among mothers directly affected by CZS.

Regarding reports of domestic violence, it is worth noting that there are no studies in the literature on the prevalence of domestic violence in mothers of children with disabilities. However, a study carried out in the capital of Espírito Santo by Leite et al.^14^ demonstrated a higher prevalence of physical partner violence experienced by women with a similar social profile as the mothers in the present study. Therefore, besides the social issues inherent in a society with a predominantly macho culture, socioeconomic factors can make women more vulnerable to violence committed by intimate partners.

Being blamed for becoming infected with Zika during pregnancy and causing CZS in the baby, either by their partners or health professionals—as reported by M8 and M6, respectively—highlights further gender inequality in the outbreak in Brazil. This inequality may originate from Brazil's patriarchal culture in which women are responsible for becoming pregnant and subsequently for any complications. Similarly, health professionals blamed women for not preventing Zika infection by not avoiding pregnancy during the outbreak and forcing the use of repellents, curtains, and even long‐sleeved clothing to prevent mosquito bites, but did not mention the role of men in the transmission of the virus or family planning.

Given the information on prevention provided by the government and the Brazilian media and the procedures they are expected to follow, women have commonly been held responsible for prevention. Thus, becoming contaminated by the virus generated a sense of guilt in the women.^24^

Blaming women for contracting Zika is erroneous given that the virus can also be transmitted sexually. Lack of information and guidance about the role of men in the prevention of Zika infection reinforces the idea that women are primarily responsible for preventing transmission. Men and boys are pivotal in preventing Zika transmission, preventing unplanned pregnancy, and ensuring that children with CZS have the best access to services and a good quality of life. Brazilian authorities at all levels should take steps to ensure that policies aimed at preventing unplanned pregnancies, Zika infection, and other sexually transmitted infections, and caring for children with CZS do not reinforce harmful gender inequities regarding male and female responsibilities, relationships, and family life.^25^

Receiving a diagnosis of fetal malformation is a difficult moment for the whole family, often causing shock, fear, and denial.^26^ The earlier the diagnosis, the less likely the risk of irreparable damage to the mother–baby relationship since there is more time for a woman and her family to adapt to the new reality.^27^, ^28^, ^29^ In the present study, many women were diagnosed at an advanced stage of pregnancy or even during birth, which heightened the shock of receiving the news.

Lack of preparation of health teams and absence of protocols and robust manuals are thought to have contributed to late diagnosis and poor investigation of suspected CZS cases, creating fear and contributing to mothers’ suffering at the onset of the outbreak.^30^ The poor ability of health professionals to communicate news of the malformation may have heightened the pain and despair of women in the present study and influenced their reaction to the situation.

In a recent Human Rights Watch survey of women who received news of a child's developmental anomaly at birth, their experience of how they were treated by doctors and nurses had a profound psychosocial impact on many women.^25^ These mothers said that the first few hours and days after the birth of their babies were marked by great anxiety, uncertainty, and doubt, but many of them did not receive complete diagnostic information or psychological support or counseling to help them cope with the news.^25^

Other studies have also revealed the lack of preparation of health professionals at the time of CZS diagnosis.^24^ It is believed that during the first wave of CZS, health professionals did not want to take responsibility for the diagnosis.^27^ Therefore, effective communication, interpersonal skills, and humanization of care must permeate the actions of health professionals.

After the birth of their children, women in the present study became the primary caregiver. Several authors have reported that in cases of children with special needs, mothers are the main caregivers, centralizing activities in the home, as well as caring for the child.^31^, ^32^, ^33^ With the burden of care focused on them, many women lose their personal identity, especially those with no support network. Due to the lack of time to perform other activities, women end up withdrawing and isolating themselves from leisure and social tasks, increasing their risk of mental illness.^34^ Mothers may find it difficult to trust others to care for their child in their absence, which can lead to psychological illness as well as physical exhaustion.^21^

Freitas et al.^35^ evaluated the impact on families of children diagnosed with microcephaly caused by the Zika virus. An impact scale revealed high scores for areas where there was greatest impact on the family, such as difficulty finding a reliable person to take care of the child, no one to understand the burden carried, and thoughts surrounding not having any more children because of an existing child's health problems.^35^

Another area that increased the suffering of women in the present study was prejudice and stigma. Insensitivity and lack of social support meant that women did not want to “share the moment” with the community which, according to Osório and Valle,^36^ is a risk factor for the process of biopsychosocial illness.

In the face of the stigma surrounding children with special needs, psychological support and the help of social workers is recommended to parents. Guaranteeing this assistance in the Unified Health System (SUS) is a particular challenge in Brazil.^37^

Many mothers of children with CZS regularly face insensitive and uninformed questions and comments about their babies. The cumulative effect of several small incidents weighs on these mothers and contributes to mental illness and social isolation.^25^

In addition to the burden of being sole caregiver, mothers face difficulties when they live far from the capital city and must commute to seek treatments for CZS. A study from Pernambuco showed the unreliability of or lack of transport from the countryside to treatment centers, in line with the findings of the present study.^38^ A similar situation in the report from Human Rights Watch describes how mothers struggle to access services for their children due to centralization of health service providers in urban areas, irregular transportation, and government bureaucracy.^25^

In conclusion, the repercussions of CZS on the lives of women in the present study were a huge burden, with important markers of social inequality and poverty revealed in the mothers’ reports. Gender inequality also marked the epidemic, along with an unequal burden of childcare, experiences of stigma, and exhausting therapeutic itineraries in search of medical care for their children.

The epidemic has created additional struggles in the lives of already vulnerable women. Brazil's fragile public policies on women's health do not reach those affected; therefore, robust public policies to support these mothers need effective implementation given that babies born with CZS need long‐term care and support. Public policies are needed to reduce working hours, provide access to daycare centers, and create better infrastructure, along with social policies that encourage sharing of domestic tasks and care between men and women. Psychological support for mothers and other family members must be implemented, as well as effective training of health professionals for informed and sensitive communication throughout pregnancy and postpartum. The Brazilian government should provide ongoing support to the necessary services so that children affected by CZS and their families may live with dignity.

## AUTHOR CONTRIBUTIONS

PSSF, ECAB, and ELNM designed and conducted the survey. PSSF, ELNM, HJSM, GBS, LCXLL, CMMS, ARLA, and ECAB conducted the data review and wrote the article. All authors analyzed and interpreted the data, critically reviewed the content, and approved the final version.

## CONFLICTS OF INTEREST

The authors have no conflicts of interest.
